# Reduced expressions of apoptosis-related proteins TRAIL, Bcl-2, and TNFR1 in NK cells of juvenile-onset systemic lupus erythematosus patients: relations with disease activity, nephritis, and neuropsychiatric involvement

**DOI:** 10.3389/fimmu.2024.1327255

**Published:** 2024-03-18

**Authors:** Bernadete L. Liphaus, Simone C. Silva, Patrícia Palmeira, Clovis A. Silva, Claudia Goldenstein-Schainberg, Magda Carneiro-Sampaio

**Affiliations:** ^1^ Laboratory of Medical Investigation, Faculdade de Medicina, Universidade de São Paulo, São Paulo, Brazil; ^2^ Pediatric Rheumatology Unit, Instituto da Criança, Faculdade de Medicina, Universidade de São Paulo, São Paulo, Brazil; ^3^ Disciplina de Reumatologia, Hospital das Clínicas, Faculdade de Medicina, Universidade de São Paulo, São Paulo, Brazil

**Keywords:** anti-dsDNA, apoptosis, Bcl-2, Juvenile SLE, NK cells, nephritis, SLEDAI-2K score, TRAIL

## Abstract

**Background:**

Lupus pathogenesis is mainly ascribed to increased production and/or impaired clearance of dead cell debris. Although self-reactive T and B lymphocytes are critically linked to lupus development, neutrophils, monocytes, and natural killer (NK) cells have also been implicated. This study assessed apoptosis-related protein expressions in NK cells of patients with juvenile-onset systemic lupus erythematosus (jSLE) and relations to disease activity parameters, nephritis, and neuropsychiatric involvement.

**Methods:**

Thirty-six patients with jSLE, 13 juvenile dermatomyositis (JDM) inflammatory controls, and nine healthy controls had Fas, FasL, TRAIL, TNFR1, Bcl-2, Bax, Bim, and caspase-3 expressions in NK cells (CD3−CD16+CD56+) simultaneously determined by flow cytometry. Disease activity parameters included Systemic Lupus Erythematosus Disease Activity Index 2000 (SLEDAI-2K) score, erythrocyte sedimentation rate, C-reactive protein level, anti-double strain DNA antibody level, complement fractions C3 and C4 levels.

**Results:**

Patients with jSLE had a profile of significantly reduced expression of TRAIL, Bcl-2, and TNFR1 proteins in NK cells when compared to healthy controls. Similar profile was observed in patients with jSLE with active disease, positive anti-dsDNA, nephritis, and without neuropsychiatric involvement. Patients with jSLE with positive anti-dsDNA also had reduced expression of Bax in NK cells when compared healthy controls and to those with negative anti-dsDNA. Yet, patients with jSLE with negative anti-dsDNA had reduced mean fluorescence intensity (MFI) of Bim in NK cells compared to healthy controls. Patients with jSLE with nephritis also had reduced MFI of Fas in NK cells when compared to those without nephritis. In addition, in patients with jSLE, the proportion of FasL-expressing NK cells directly correlated with the SLEDAI-2K score (rs = 0.6, p = 0.002) and inversely correlated with the C3 levels (rs = −0.5, p = 0.007). Moreover, patients with jSLE had increased NK cell percentage and caspase-3 protein expression in NK cells when compared to JDM controls.

**Conclusion:**

This study extends to NK cells an altered profile of TRAIL, Bcl-2, TNFR1, Fas, FasL, Bax, Bim, and caspase-3 proteins in patients with jSLE, particularly in those with active disease, positive anti-dsDNA, nephritis, and without neuropsychiatric involvement. This change in apoptosis-related protein expressions may contribute to the defective functions of NK cells and, consequently, to lupus development. The full clarification of the role of NK cells in jSLE pathogenesis may pave the way for new therapies like those of NK cell–based.

## 1 Introduction

Juvenile-onset systemic lupus erythematosus (jSLE) is a multisystem autoimmune disorder with a relapsing-remitting course, a high risk of nephritis and neuropsychiatric involvement, a more active disease, and an increased mortality ratio when compared to adult SLE (1). There is evidence that jSLE has stronger genetic background and interferon (IFN) signature (1–3). Although self-reactive T and B lymphocytes are critically linked to lupus development, others cells as neutrophils, monocytes, and natural killer (NK) cells have also been implicated (1, 4–6). Lupus pathogenesis is mainly ascribed to increased production and/or inefficient removal of dead cell debris (1, 4, 7–12). In this regard, our group demonstrated that patients with jSLE have altered expressions of the apoptosis-related proteins Fas and Bcl-2 in lymphocytes and monocytes, as well as altered sFas, sTRAIL, sFasL, and sMer levels, which related to disease activity and/or nephritis (13–17).

NK are innate, CD3-negative, cells that comprise 5% to 15% of peripheral blood mononuclear components and lack antigen specificity (5). NK cells detect and have critical cytolytic effector role in response to intracellular pathogens and transformed or stressed cells (5). NK cell cytotoxicity is regulated by a series of cytokines including IFN-α and IFN-γ (5, 18). Moreover, NK cell release of cytokines modulates not only the innate immune response but also the proliferation of helper T cells (5). Therefore, NK cell actions must be carefully regulated to prevent: 1. inappropriate apoptosis and tissue damage, 2. augmented cytokine release, 3. dysregulated adaptive immune response, and 4. persistent T-cell activation (5, 18). The best characterized inhibitory and activating receptors in NK cells are those of the NKG2, NCRs, and the KIR families, whereas the receptors related to apoptosis are poorly understood (5, 18).

Studies show a reduced absolute number and frequency of NK cells as well as impaired cytotoxic effect and defects of NK differentiation in SLE and other autoimmune diseases (5, 19–21). In addition, altered proportions of NK cells have been related to lupus nephritis, thrombocytopenia, and disease activity (19, 20, 22, 23). Defective NK cell cytolysis was also observed in jSLE (22). Furthermore, depletion of NK cells in mice led to the development of autoantibody-secreting B lymphocytes (5, 18, 24).

Reports evaluating apoptosis-related proteins in NK cells are sparse (5, 18, 25). One study showed increased FasL expression in NK cells of adult patients with SLE, and another reported higher Fas expression by NK cells of multiple sclerosis patients (26, 27).

Therefore, this study simultaneously assessed the expression of both inducing (Fas, TRAIL, TNFR1, Bax, and Bim) and inhibitory (Bcl-2) apoptosis-related proteins from the extrinsic (Fas, FasL, TRAIL, and TNFR1), the intrinsic (Bcl-2, Bax, and Bim), and the caspase-dependent (caspase-3) pathways in peripheral NK cells of patients with jSLE and investigated the relations with disease activity parameters, nephritis and neuropsychiatric involvement.

## 2 Methods

### 2.1 Patients and controls

Patients and controls were consecutively included in this cross-sectional study, comprising 36 patients with jSLE, 13 juvenile dermatomyositis (JDM) inflammatory controls, and nine sex- and age-matched non-related children without inflammatory and/or autoimmune disease (healthy controls). All patients and controls were followed at the hospital pediatric rheumatology unit. Informed assent/consent was obtained from parents and participants after approval by the local ethics committee. All patients fulfilled respective classification criteria and were younger than 18 years at disease onset (28–30). On enrollment, individuals with suspicion of infection were excluded.

Medical records were revised for patients’ demographic, clinical, laboratory, and current treatment data. Nephritis and neuropsychiatric involvement were defined according to lupus criteria (28, 29). Disease activity parameters included erythrocyte sedimentation rate (ESR; by Westergren method), C-reactive protein level (CRP; by nephelometry), complement fractions C3 and C4 levels (by nephelometry), anti-dsDNA antibody level (by Enzyme-linked Immunosorbend Assay (ELISA) assay), and the Systemic Lupus Erythematosus Disease Activity Index 2000 (SLEDAI-2K) score (31). Arbitrarily, the active disease was defined as SLEDAI-2K score ≥ 4. Patients and controls data are described in the **Supplementary Table.**

### 2.2 Staining reagents

The following surface and intracellular monoclonal anti-human antibodies were used: PE-Cy7 anti-CD3, APC anti-CD56, PERCP anti-CD56, APC-Cy7 anti-CD16, V450 anti-Fas/CD95, PE anti-FasL, APC anti-TRAIL, PERCP anti-TNFR1/CD120, V450 anti-Bcl-2, FITC anti-Bax, PE anti-Bim, and FITC anti-caspase-3. Isotype controls were included in all experiments (PERCP IgG1, FITC IgG1, PE IgG1, and V450 IgG1).

### 2.3 NK cell preparation

Immediately after peripheral venous blood was collected in EDTA tubes, 0.5 ml was placed in each tube containing lysis buffer (BD Lysing buffer) for 30 min at room temperature. Cells were then washed two times and resuspended in a staining buffer to obtain 2 × 10 (6) cells in 100 μL. The cell suspension was placed in a 96-well plate, and 100 μL of diluted membrane antibodies (CD3, CD56, CD16, Fas, FasL, TRAIL, and TNFR1/CD120) were added and incubated for 30 min at 4°C in dark. The plate was washed, the supernatant was discarded by inversion, and cells were resuspended in 300 μL of staining buffer. For intracellular staining, 100 μL of Cytofix/Cytoperm was added per well and incubated for 30 min at 4°C in dark. Afterward, 100 μL of PBS was added and centrifuged at 1,200 rpm at 4°C for 5 min, and 130 μL of 1% paraformaldehyde PBS 0.5% Tween 20 was added and incubated for 30 min at room temperature in dark. Then, 100 μL of diluted intracellular antibodies (Bcl-2, Bax, Bim, and caspase-3) were added and incubated for 2 h at room temperature in dark. After washing, cells were resuspended in 300 μL of staining buffer.

### 2.4 Flow cytometry analysis

The eight-color analysis was performed with the FACS LRS II Fortessa flow cytometer (BD Biosciences) and the FlowJo™. NK cell proportions were determined in the lymphocyte gate. To exclude T cells and monocytes, NK cells were analyzed by the selection of CD3-negative cells. NK cells were detected on the CD16 versus CD56 expression plot. This gating strategy detected double-positive CD16+ CD56+ NK cells. Considering that classifying NK cells into CD56^dim^ or CD56^bright^ subsets does not necessarily define the functional phenotype in different physiological settings (5), we chose not to assess the NK cell subpopulations. Apoptosis-related protein expressions are shown by percentages of NK cells expressing the respective protein and by the relative quantitative densities of each protein in the NK cells measured as mean fluorescence intensity (MFI). Representatives gating strategy and flow cytometry for each apoptosis-related protein expression in NK cells of the three groups are shown in the **Supplementary Figure**.

### 2.5 Statistical analysis

According to data normality, tested by the D’Agostino-Pearson normality test, group comparisons were performed using the nonparametric KruskalWallis test, with Dunn’s multiple comparisons *post-hoc* test. Continuous variables were correlated using Spearman’s rank test. Analyses were carried out using GraphPad Prism version 7.0 Software for Windows (GraphPad Software Inc., San Diego, CA, USA). Data are shown as number and percentage or as mean ± standard deviation (SD) or as median and range. Only non-zero expression results were included in the analysis. All statistical tests were performed considering a significant level of p < 0.05.

## 3 Results

### 3.1 Demographic, clinical, and disease activity features from patients and controls

The 36 patients with jSLE (31 girls) had median age upon enrollment of 15.8 years, median disease duration of 4.6 years, and median age at disease onset of 10.9 years. Twenty-nine patients with jSLE had nephritis, and 37.9% underwent renal biopsy. Nine patients with jSLE had neuropsichiatric involvement. In terms of disease activity, 11 (30.6%) patients with jSLE had SLEDAI-2K score ≥ 4, and 19 patients had SLEDAI-2K score equal to zero. The median SLEDAI-2K score was zero, median ESR was 20.5 mm/h, median CRP was 0.9 mg/dL, and median anti-dsDNA antibody level was 46.4 IU/mL. Twenty-three patients with jSLE had positive anti-dsDNA. All patients with jSLE were taking hydroxychloroquine associated with one or more of azathioprine, mycophenolate mofetil, methotrexate, cyclosporine, and prednisone. Fifteen (41.7%) patients with jSLE were taking prednisone. The 13 patients with JDM (eight girls) had median age upon enrollment of 14.4 years, median disease duration of 5.6 years, and median age at disease onset of 6.9 years. JDM controls had median ESR of 13.0 mm/h and median CRP of 3.4 mg/dL. Five (38.5%) patients with JDM were taking prednisone. The nine healthy controls (eight girls) had median age upon enrollment of 14.2 years, median ESR of 8.5 mm/h, and median CRP of 0.3 mg/dL. Additional patients and controls data are summarized in the **Supplementary Table.**

### 3.2 Frequency of peripheral NK cells

**Figure 1:**
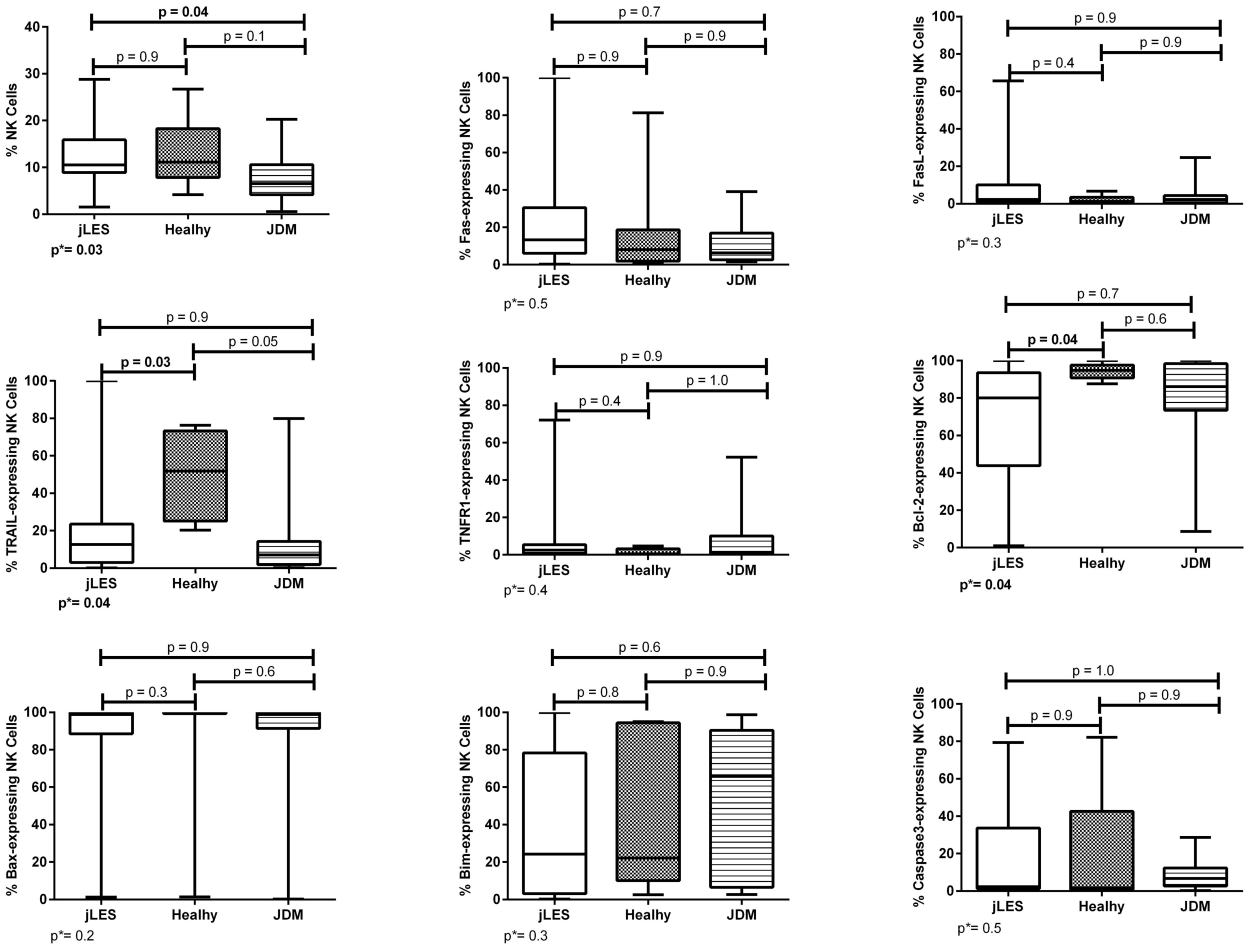
Percentage of NK cells and proportions of apoptosis-related proteins in NK cells of patients with juvenile-onset systemic lupus erythematosus (jSLE), juvenile dermatomyositis (JDM) inflammatory controls, and healthy controls. Results presented as median, range, and 25th–75th percentiles. p* represents the three group comparisons, and p shows the adjusted values.

NK cell percentage differed comparing the three groups (p = 0.03). Then, patients with jSLE had significantly increased NK cell percentage when compared to JDM controls [median: 10.5% (range: 1.5–28.8) vs. median: 6.5% (range: 0.5–20.3), p = 0.04], whereas NK cell percentage of patients with jSLE was like healthy controls [median: 10.5% (range: 1.5–28.8) vs. median: 11.1% (range: 4.2–26.7), p = 0.9], as shown in **Figure 1**.

### 3.3 Expressions of apoptosis-related proteins

**Figure 2:**
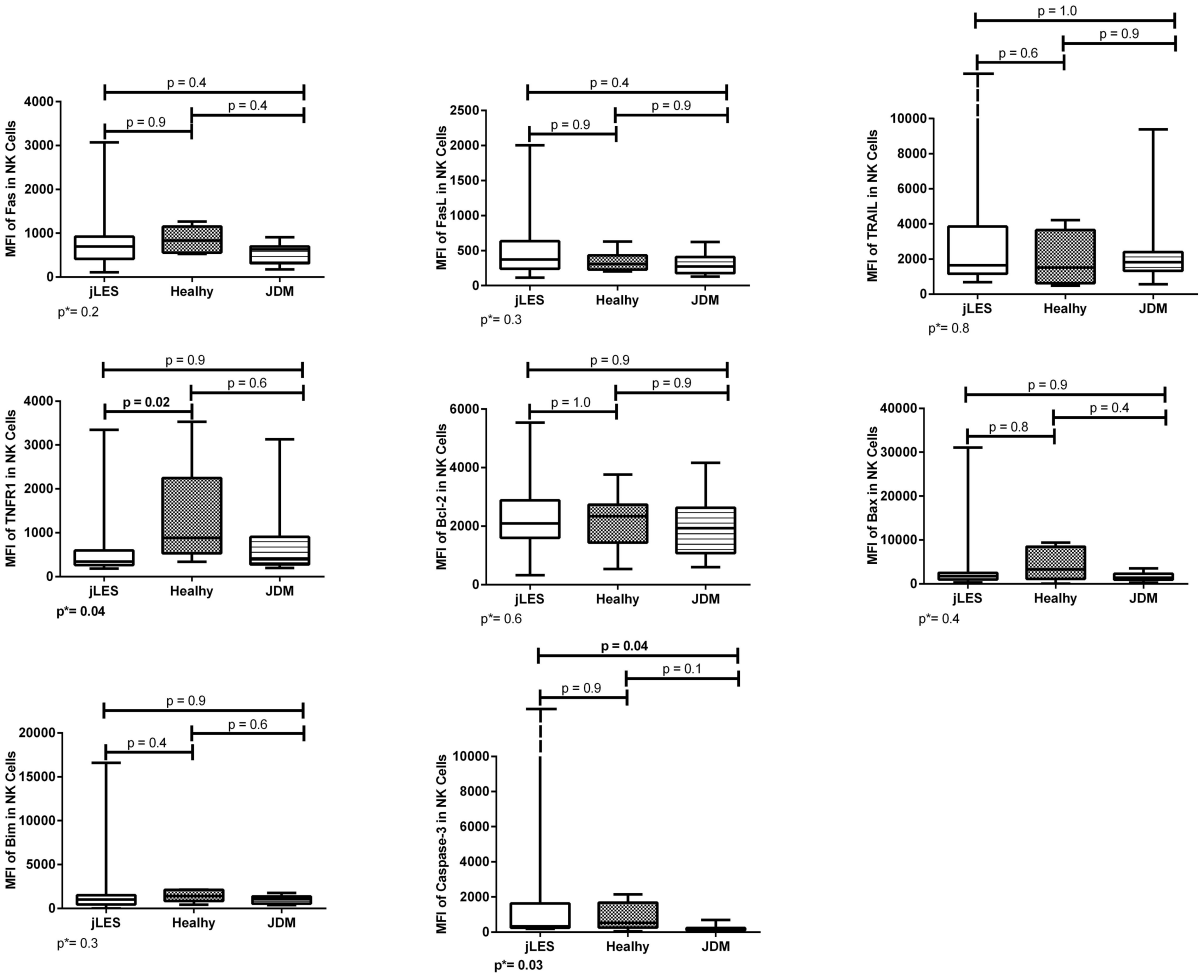
Densities (MFI) of apoptosis-related proteins in NK cells of patients with juvenile-onset systemic lupus erythematosus (jSLE), juvenile dermatomyositis (JDM) inflammatory controls, and healthy controls. Results presented as median, range, and 25th–75th percentiles. p* represents the three group comparisons, and p shows the adjusted values.

Patients with jSLE had significantly reduced proportions of TRAIL [median: 12.6% (range: 0.2–100.0) vs. median: 51.9% (range: 20.3–76.3), p = 0.03] and Bcl-2–expressing NK cells [median: 80.0% (range: 1.0–100.0) vs. median: 94.8% (range: 87.6–100.0), p = 0.04] as well as reduced density of TNFR1 protein in NK cells [median MFI: 343.0 (180.0–3,346.0) vs. median MFI: 886.0 (337.0–3,532.0), p = 0.02] when compared to healthy controls. Patients with jSLE also had significantly increased density of caspase-3 protein in NK cells [median MFI: 325.0 (range: 186.0–12,200.0) vs. median MFI: 529.0 (range: 42.0–2,157.0), p=0.04] when compared to JDM controls, as shown in **Figures 1**, **2**. Yet, when comparing the three groups, no difference of apoptosis-related protein expression was pointed between patients with JDM and healthy controls. Thus, we hypothesize whether the altered apoptosis-related protein expressions of patients with jSLE could differ according to disease activity or the clinical manifestation.

### 3.4 Relations with disease activity parameters

**Figure 3:**
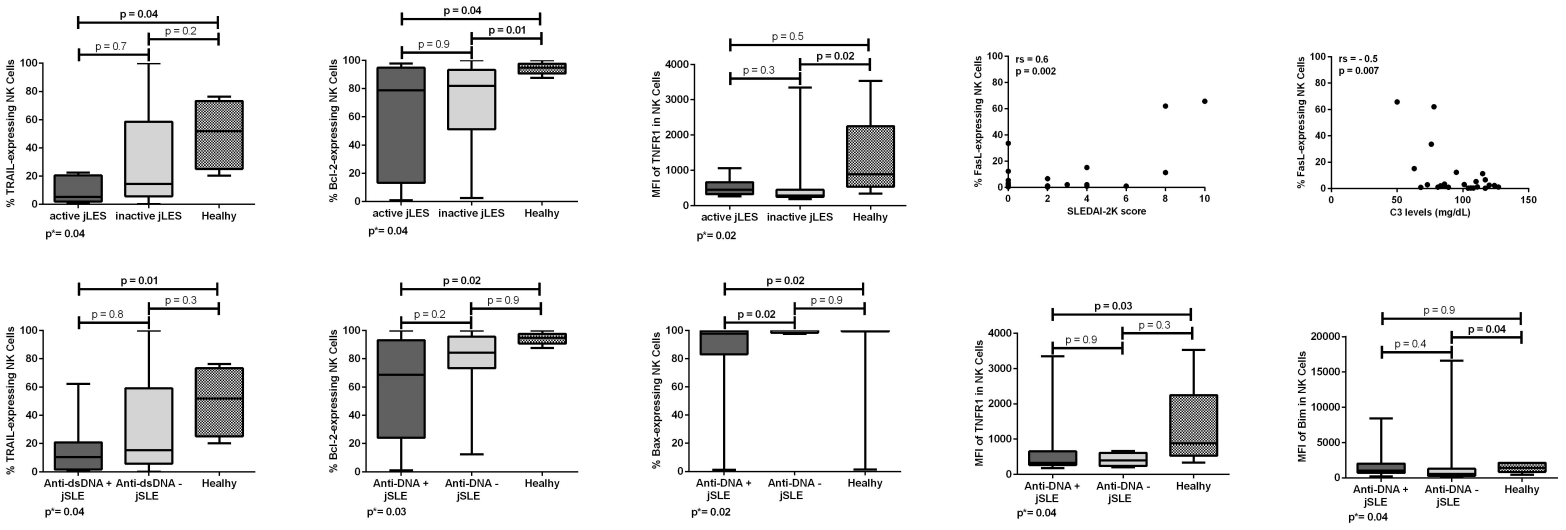
Proportions and densities (MFI) of apoptosis-related proteins in NK cells of patients with juvenile-onset systemic lupus erythematosus (jSLE) according to disease activity or dsDNA positivity, and healthy controls. Correlations of proportions of apoptosis-related proteins in NK cells of patients with jSLE and the SLEDAI-2K score and C3 levels. Results presented as median, range, and 25th–75th percentiles. p* represents the three group comparisons, and p shows the adjusted values.

Patients with jSLE with active disease (SLEDAI-2K score ≥ 4) had significantly reduced proportion of TRAIL [median: 5.4% (range: 0.8–22.5) vs. median: 51.9% (range: 20.3–76.3), p = 0.04] and Bcl-2–expressing NK cells [median: 78.8% (range: 1.0–97.8) vs. median: 94.8% (range: 87.6–100.0), p = 0.04] when compared to healthy controls. Patients with jSLE with inactive disease had significantly reduced proportion of Bcl-2–expressing NK cells [median: 81.9% (range: 2.5–100.0 vs. median: 94.8% (range: 87.6–100.0), p = 0.01] as well as reduced MFI of TNFR1 protein in NK cells [median MFI: 282.0 (180.0–3,346.0) vs. median MFI: 886.0 (337.0–3,532.0), p = 0.02] when compared to healthy controls. Patients with jSLE with active disease had similar NK cell percentage when compared to healthy controls [median: 9.6% (range: 1.5–24.3) vs. median: 11.1% (range: 4.2–26.7), p = 0.2]. Moreover, in patients with jSLE, the proportion of FasL-expressing NK cells directly correlated with the SLEDAI-2K score (rs = 0.6, p = 0.002) and inversely correlated with the C3 levels (rs = −0.5, p = 0.007). Significant comparisons and correlations are shown in **Figure 3**.

Patients with jSLE with positive anti-dsDNA had significantly reduced proportion of TRAIL [median: 10.4% (range: 0.8–62.2) vs. median: 51.9% (range: 20.3–76.3), p = 0.01], Bcl-2 [median: 68.6% (range: 1.0–100.0) vs. median: 94.8% (range: 87.6–100.0), p = 0.02], and Bax-expressing NK cells [median: 97.8% (range: 1.3–100.0) vs. median: 99.9% (range: 1.4–100.0), p = 0.02] as well as reduced density of TNFR1 protein in NK cells [median MFI: 325.0 (180.0–3,346.0) vs. median MFI: 886.0 (337.0–3,532.0), p = 0.03] when compared to healthy controls. Patients with jSLE with positive anti-dsDNA also had significantly reduced proportion of Bax-expressing NK cells [median: 97.8% (range: 1.3–100.0) vs. median: 100.0% (range: 97.5–100.0), p = 0.02] when compared to those with negative anti-dsDNA. Yet, patients with jSLE with negative anti-dsDNA had significantly reduced MFI of Bim protein in NK cells [median MFI: 549.0 (2.9–16,600.0) vs. median MFI: 1,414.0 (428.0–2,136.0), p = 0.04] when compared to healthy controls. Patients with jSLE with positive anti-dsDNA had similar NK cell percentage when compared to healthy controls [median: 10.2% (range: 3.6–28.8) vs. meidan: 11.1% (range: 4.2-26.7), p = 0.7]. Significant comparisons are shown in **Figure 3**. Intriguingly, anti-dsDNA levels did not correlate either with apoptosis-related protein proportions or densities (data not shown).

### 3.5 Relations with nephritis

**Figure 4:**
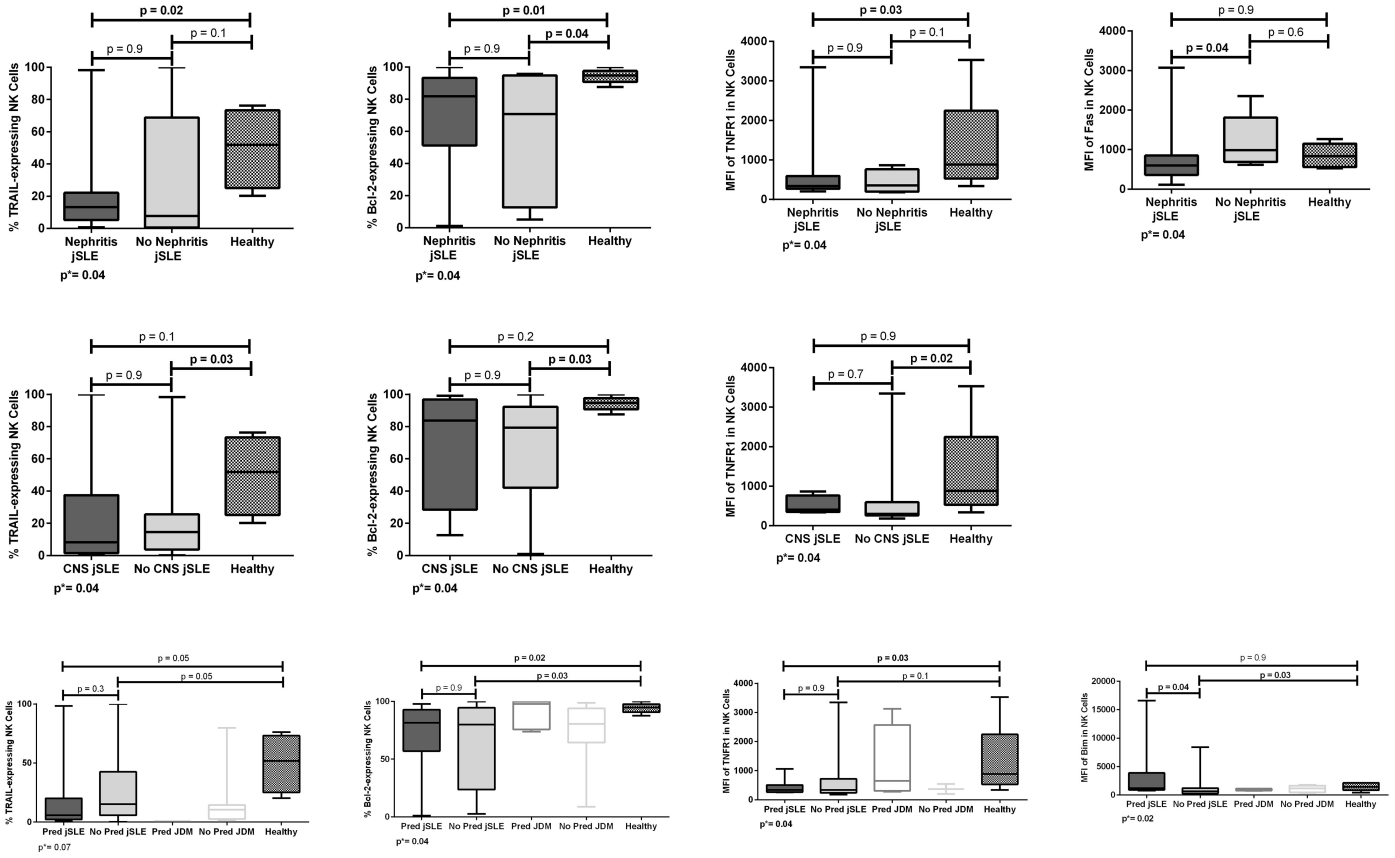
Proportions and densities (MFI) of apoptosis-related proteins in NK cells of patients with juvenile-onset systemic lupus erythematosus (jSLE) according to renal or central nervous system (CNS)/neuropsychiatric involvement, or prednisone treatment, and healthy controls. Results presented as median, range, and 25th–75th percentiles. p* represents the three group comparisons, and p shows the adjusted values.

Patients with jSLE with nephritis had significantly reduced proportion of TRAIL [median: 13.3% (range: 0.8–98.3) vs. median: 51.9% (range: 20.3–76.3), p = 0.02] and Bcl-2–expressing NK cells [median: 81.8% (range: 1.0–100.0) vs. median: 94.8% (range: 87.6–100.0), p = 0.01] as well as reduced MFI of TNFR1 protein in NK cells [median MFI: 343.0 (206.0–3,346.0) vs. median MFI: 886.0 (337.0–3,532.0), p = 0.03] when compared to healthy controls, and reduced MFI of Fas protein in NK cells [median MFI: 603.0 (113.0–3,072.0) vs. median: 989.0 (618.0–2,355.0), p = 0.04] when compared to those without nephritis. Patients with jSLE without nephritis had significantly reduced proportion of Bcl-2–expressing NK cells [median: 70.7% (range: 5.0–95.8) vs. median: 94.8% (range: 87.6–100.0), p = 0.04] when compared to healthy controls. Patients with jSLE with nephritis had similar NK cell percentage when compared to healthy controls [median: 10.2% (range: 1.5–24.3) vs. median: 11.1% (range: 4.2–26.7), p = 0.5]. Significant comparisons are shown in **Figure 4**.

### 3.6 Relations with neuropsychiatric involvement

Patients with jSLE without neuropsychiatric involvement had significantly reduced proportion of TRAIL [median: 14.5% (range: 0.2–98.3) vs. median: 51.9% (range: 20.3–76.3), p = 0.03] and Bcl-2–expressing NK cells [median: 79.4% (range: 1.0–100.0) vs. median: 94.8% (range: 87.6–100.0), p = 0.03] as well as reduced density of TNFR1 protein in NK cells [median MFI: 303.0 (180.0–3,346.0) vs. median MFI: 886.0 (337.0–3,532.0), p = 0.02] when compared to healthy controls. Patients with jSLE with neuropsychiatric involvement had similar NK cell percentage when compared to healthy controls [median: 11.8% (range: 3.9–16.8) vs. 11.1% (range: 4.2–26.7), p = 0.8]. Significant comparisons are shown in **Figure 4**.

### 3.7 Relations with corticosteroid treatment

Fifteen (41.7%) patients with jSLE and 5 (38.5%) patients with JDM were taking prednisone. We performed this analysis to clarify possible confounding caused by treatment. NK cell percentage and apoptosis-related protein expressions did not differ when comparing the five groups as well as when comparing patients with JDM taking or not prednisone and healthy controls (data not shown). Otherwise, patients with jSLE taking prednisone had significantly reduced proportions of Bcl-2–expressing NK cells [median: 81.5% (range: 1.0–97.8) vs. median: 94.8% (87.6–100.0), p = 0.02] and reduced density of TNFR1 protein in NK cells [median: MFI 337.5 (257.0–1,061.0) vs. median MFI: 886.0 (337.0–3,532.0), p = 0.03] when compared to healthy controls as well as increased density of Bim protein in NK cells [median MFI: 1,182.0 (761.0–16,600.0) vs. median MFI: 643.0 (2.9–8,411.0) p = 0.04] when compared to those not taking prednisone. Patients with jSLE not taking prednisone had significantly reduced proportion of Bcl-2–expressing NK cells [median: 80.0% (range: 2.5–100.0 vs. median: 94.8% (range: 87.6–100.0), p = 0.03] and reduced MFI of Bim protein in NK cells [median MFI: 643.0 (2.9–8,411.0) vs. median MFI: 1,414.0 (428.0–2,136.0), p = 0.03] when compared to healthy controls. Patients with jSLE taking prednisone had NK cell percentage similar to healthy controls [median: 10.2% (range: 1.5–20.9) vs. median: 11.1% (range: 4.2–26.7), p=0.5]. Significant comparisons are shown in **Figure 4**.

## 4 Discussion

To the best of our knowledge, this is the first study to simultaneously assess the expression of eight apoptosis-related proteins in NK cells of patients with jSLE. We found that NK cells of patients with jSLE have a profile characterized by reduced TRAIL, Bcl-2, and TNFR1 expressions when compared to those of healthy controls and increased caspase-3 expression when compared to those of JDM controls. Reduced TRAIL and Bcl-2 expressions were also observed in patients with jSLE with active disease. In addition, FasL expression correlated directly with the SLEDAI-2K score and inversely with C3 levels. Reduced TRAIL, Bcl-2, TNFR1, and Bax expressions were observed in patients with positive anti-dsDNA. Otherwise, patients with jSLE with negative anti-dsDNA had reduced Bim expression. Reduced TRAIL, Bcl-2, TNFR1, and Fas expressions occurred in patients with nephritis. Moreover, reduced TRAIL, Bcl-2, and TNFR1 expressions were also observed in patients without neuropsychiatric involvement. Yet, patients with jSLE had the frequency of NK cells similar to healthy controls and increased when compared to JDM controls.

The development of jSLE has been linked to changes in the expression of proteins involved in apoptosis, such as Fas and Bcl-2, in lymphocytes, monocytes, and neutrophils (7–10, 13–17). This raises the question of whether other proteins or cells involved in apoptosis could also play a role in jSLE pathogenesis. This study found that the profile of apoptosis-related proteins in patients with jSLE is complex; includes other proteins like TRAIL, TNFR1, FasL, Bax, Bim, and caspase-3, as well as other cells like NK cells; and diverge according to disease activity and the organ involved.

The protective role of NK cells in autoimmunity has been related to its downregulation of autoreactive adaptive immune responses (5, 18). In this way, NK cell numeric and/or functional defects have been observed in patients with autoimmune diseases (5, 20, 21, 27). Phenotypic alterations, such as increased CD69 and CD86 expressions in patients’ NK cells, suggest a dysfunctional state (5). However, the mechanisms supporting these abnormalities are still not definitive (5). Studies demonstrated the accumulation of NK cells in affected tissues of autoimmune patients, indicating a possible dysregulated NK cell apoptosis (5, 18). NK cells induce apoptosis of target cells via the caspase-dependent pathway linking up FasL, TNF, and TRAIL receptors (5, 18, 25). Another way, the NK cells’ apoptosis is mediated by both Fas–FasL interactions and via CD16 engagement (18, 25). In fact, all these apoptosis-related protein expressions were, herein, observed to be altered in patients with jSLE.

Studies evaluating the absolute number and frequency of NK cells in patients with lupus are still controversial (5, 19, 26). This study shows that patients with jSLE have an increased percentage of NK cells when compared to JDM controls. We speculate whether the altered expressions of apoptosis-related proteins observed herein may be influencing the result of the number of NK cells reported by different studies. We also reflect the similar percentage of NK cells among patients with jSLE, and healthy controls could not be attributed to for not discriminating NK cells CD56^dim^ from CD56^bright^ subset (5, 23, 32). Nonetheless, according to our results, the similar percentage of NK cells of patients with jSLE and healthy controls cannot be attributed to disease activity or prednisone treatment.

NK cells from patients with lupus also present altered functions, namely. defective cytolysis (5, 20, 22, 23). This study did not directly analyze NK cells’ cytolysis. However, we speculate whether the killing defect of NK cells may be related to the reduced expressions of apoptosis-related proteins observed herein because it could not be entirely explained by cytokines production in an earlier study (20).

Previous studies report constitutively lower Fas, FasL, and Bcl-2 expressions in freshly isolated NK cells when compared to those in T lymphocytes (5, 18, 25). We observed herein that patients with jSLE had Fas and FasL expressions similar to controls, as well as reduced TRAIL and Bcl-2 expressions. These findings diverge from our previous observations of increased expressions in helper and cytotoxic T cells and B lymphocytes and reduced expressions in monocytes (13–15). These remarks also contrast with the higher Fas expression in NK cells of multiple sclerosis patients (27). In line, it is diverse of the significantly increased soluble Fas levels in the serum of patients with jSLE (16).

TRAIL is an apoptosis-inducing ligand by interacts with its death receptors DR4 and DR5 in monocytes, lymphocytes, and NK cells (5, 18, 33). The reduced proportion of TRAIL-expressing NK cells in patients with jSLE observed herein contrasts with previous results of increased TRAIL-expressing T lymphocytes, TRAIL mRNA expression levels, and soluble TRAIL levels reported in both patients with adult SLE and patients with jSLE (16, 33, 34). This finding suggests a decreased TRAIL-mediated apoptosis and, as a consequence, an impaired cytolysis of autoreactive cells.

As for TRAIL protein, Fas/FasL interactions are essential for activated and autoreactive immune cell deletion (7). One study showed that even patients with jSLE with inactive disease persist with cytolytic defects in NK cells (22). We doubt if this defect is related to Fas/FasL pathway because we observed their expressions in jSLE NK cells were like the healthy controls. Otherwise, a reduced expression of Fas occurred in patients with nephritis. Yet, the correlations between the proportion of FasL-expressing in NK cells and the SLEDAI score and C3 levels are in line with a previous report in adult SLE, which observed similar Fas and increased FasL expressions in NK cells of patients with active disease (26). In addition, we anteriorly observed reduced sFasL levels in patients with jSLE (16). Moreover, based on a previous study, we could speculate whether the not increased Fas-expressing NK cells may define an NK1-like phenotype in jSLE (24).

Mice lacking Bcl-2 have lymphocytes abnormally susceptible to a range of death stimuli (35). NK cells constitutively expressed low levels of Bcl-2 (5, 18, 25). Although, in this study, Bcl-2 proportions and MFI have greatly varied, the reduced proportion of Bcl-2–expressing NK cells may favor the apoptosis rate and lead to an overflow of phagocytes with apoptotic bodies. Nonetheless, this result differs from previous ones that observed increased Bcl-2 expressions in T and B lymphocytes of patients with jSLE (13–15).

Bim and Bax’s relevance to autoimmune diseases was first shown by Bim knockout mice that accumulated plasma cells and developed high titers of autoantibodies, vasculitis, and diffuse proliferative glomerulonephritis with immune complex deposition similar to SLE (34). Otherwise, single Bax knockout mice are essentially normal, which demonstrated its largely overlapping functions with Bak (34). Additional knockout mice studies demonstrated that the absence of Bcl-2 increases cell dying due to unopposed action of Bim, whereas concomitant removal of Bim abrogates this effect (35). Interestingly, patients with jSLE with positive anti-dsDNA had a significantly reduced proportion of Bax-expressing NK cells when compared to both healthy controls and patients with negative anti-dsDNA. Yet, patients with jSLE with negative anti-dsDNA had significantly reduced density of Bim protein in NK cells when compared to healthy controls. These findings open a new avenue in understanding the role of Bax, its balance with Bim, Bak, and Bcl-2 in lupus development, as well as of possible biomarkers of disease activity.

In NK cells, the caspase-3–dependent pathway can be activated not only by the extrinsic and intrinsic pathways but also by granzymes (5, 18). Caspase-3, FasL, and Bax expressions were reported to be high in lupus nephritis, especially caspase-3 and Bax in glomeruli of class IV (36). The present study shows that patients with jSLE have an increased density of caspase-3 protein in NK cells compared to JDM controls, although the same was not observed in patients with nephritis. Thus, the real role of caspase-3 in lupus pathogenesis still needs to be clarified.

Patients with jSLE with active disease had the lowest median proportion of TRAIL-expressing NK cells and those with positive anti-dsDNA had the lowest median proportion of Bcl-2–expressing NK cells. Moreover, patients with jSLE with positive anti-dsDNA had reduced TRAIL, Bax, and TNFR1 expressions in NK cells. Furthermore, the proportion of FasL-expressing NK cells correlated with the SLEDAI score and C3 levels. These findings put TRAIL, Bcl-2, Bax, and TNFR1 expressions in NK cells as also good candidates for future biomarkers for monitoring disease activity.

To shed light on the role of NK cells in lupus clinical features, we related apoptosis protein expressions with the manifestations with higher risk of morbidity and mortality. Although nephritis is one of the most serious manifestations of jSLE, it still does not have an ideal biomarker to detect early renal flare (1, 4). In this study, patients with jSLE with nephritis had reduced TRAIL, Bcl-2, TNFR1, and Fas expressions in NK cells when compared to healthy controls and to those without nephritis, confirming the great imbalance of the apoptosis process in patients with jSLE with nephritis, which favors disease development and maintenance. One or more of these protein expressions may be considered as possible nephritis flare biomarkers, mainly Bcl-2 that had the lowest proportion in patients with positive anti-dsDNA. Furthermore, patients with jSLE without neuropsychiatric involvement had reduced proportions of TRAIL and Bcl-2–expressing NK cells that may reflect the whole group results or suggest a protective role of these proteins to the central nervous system, an aspect to be better clarified.

A stronger genetic predisposition is observed in patients with early-onset (≤ 6 years of age) jSLE (1–4, 37). Nonetheless, the three patients with early-onset jSLE and the 6 patients with prepubertal disease onset (≤ 8 years of age) had apoptosis-related protein expressions similar to the whole group (data not shown) precluding additional analyses.

Corticosteroids and immunosuppressive drugs can modify both membrane and cytoplasmic expressions of apoptosis-related proteins (7–10). Thus, drug therapy interference with the observed altered expressions may not be completely ruled out. In this regard, patients with jSLE taking prednisone had results of apoptosis-related protein proportions and densities similar to the whole group, suggesting that either these were potentially influenced by the treatment or reflected the findings of active patients in need of treatment. Therefore, prospective studies on drug influence on NK cell number and/or functions are still required.

This study has some unavoidable limitations such as the single-center design, the limited sample size, its cross-sectional nature, not having the opportunity to study other apoptosis-related proteins expressed by NK cells as TWEAK and Bid, and not assessing them locally in the tissues, particularly in the kidney. In addition, apoptosis-related protein expressions displayed a high variation of proportions and MFI, which may reflect the complex scenario of analyzing NK cell phenotype.

Lastly, our findings in NK cells reinforce the importance of determining apoptosis-related protein expressions in jSLE and not extrapolating adult SLE results. In addition to this, the reduced TRAIL, Bcl-2, TNFR1, and Fas expressions in patients with nephritis favor the hypothesis that the disrupted balance of apoptosis proteins may result in increased death of both activated NK and target cells, leading to a greater offer of apoptotic bodies to the immune system. However, this same disrupted balance may result in abnormal longevity of autoreactive NK cells and, consequently, a tolerance disorder. Moreover, the dysregulated apoptosis process, observed in patients with jSLE, appears not to be limited to a particular cell type, because altered expressions of distinct apoptosis-related proteins have been observed in helper and cytotoxic T cells, B cells, monocytes, and, herein, NK cells (13–17). Furthermore, it is possible that apoptotic proteins are not primarily defective in jSLE, but rather the autoimmune process modifies their expressions and, then, the activated apoptotic mechanism contributes to disease development.

## 5 Conclusion

This study extends to NK cells an altered profile of TRAIL, Bcl-2, TNFR1, Fas, FasL, Bax, Bim, and caspase-3 proteins in patients with jSLE, particularly in those with active disease, positive anti-dsDNA, nephritis, and without neuropsychiatric involvement. This change in apoptosis-related protein expressions may contribute to the defective functions of NK cells and, consequently, to lupus development. The full clarification of the role of NK cells in jSLE pathogenesis may pave the way for new therapies like those of NK cell–based.

## Data availability statement

The original contributions presented in the study are included in the article/**Supplementary Material**. Further inquiries can be directed to the corresponding author.

## Ethics statement

The studies involving humans were approved by Instituto da Criança and Hospital das Clínicas ethics committee. The studies were conducted in accordance with the local legislation and institutional requirements. Written informed consent for participation in this study was provided by the participants’ legal guardians/next of kin.

## Author contributions

BL: Conceptualization, Data curation, Formal Analysis, Funding acquisition, Investigation, Project administration, Writing – original draft, Writing – review & editing. SS: Data curation, Methodology, Writing – original draft. PP: Data curation, Formal Analysis, Methodology, Writing – original draft. CS: Conceptualization, Investigation, Writing – original draft. C-GS: Conceptualization, Data curation, Investigation, Writing – original draft. MC-S: Conceptualization, Funding acquisition, Investigation, Writing – original draft.
