# Supplementary material for: Reduced expressions of apoptosis-related proteins TRAIL, Bcl-2, and TNFR1 in NK cells of juvenile-onset systemic lupus erythematosus patients: relations with disease activity, nephritis, and neuropsychiatric involvement
Source: Front Immunol. 2024 Mar 18;15:1327255. doi: 10.3389/fimmu.2024.1327255 (PMC10982494; doi:10.3389/fimmu.2024.1327255)
Supplement: Supplementary file 1 [file DataSheet_1.pdf]

Supplementary Table Demographic, clinical, and laboratory characteristics, current treatments and disease activity features of juvenile-onset systemic lupus erythematosus (jSLE) patients, juvenile dermatomyositis (JDM) inflammatory controls, and healthy controls.

	**jSLE** (n=36)	**JDM** (n=13)	**Healthy** (n=9)
Demographic data	Mean ± SD (range) or Number (%)		
Age upon enrollment, years	15.1 ± 2.6 (7.1 – 20.3)	13.4 ± 3.6 (7.6 – 17.5)	14.7 ± 4.4 (8.4 – 22.9)
Age at disease onset, years	10.8 ± 3.1 (4.1 – 18.7)	7.2 ± 2.9 (1.8 – 12.7)	-
Disease duration, years	4.7 ± 2.9 (0.2 – 11.4)	6.8 ± 4.7 (1.1 – 16.2)	-
Female / Male	31(86.1) / 5(13.9)	8(61.5) / 5(38.5)	8(88.9) / 1(11.1)
Clinical Manifestation	Number (%)		
Cutaneous	36 (100.0)	12 (92.3)	-
Hematological	32 (88.9)	1 (7.7)	-
Musculoskeletal	33 (91.7)	13 (100.0)	-
Neuropsychiatric	9 (25.0)	1 (7.7)	-
Nephritis	29 (80.6)	1 (7.7)	-
Pulmonary	13 (36.1)	5 (38.5)	-
Cardiovascular	14 (38.9)	-	-
Ophthalmological	5 (13.8)	3 (23.1)	-
Gastrointestinal	8 (22.2)	5 (38.5)	-
Histological class	Number (%)		
Renal biopsy	11/29 (37.9)	-	-
Class III / IV / V	2 / 3 / 6	-	-
Treatment	Number (%)		
Prednisone	15 (41.7)	5 (38.5)	-
Antiinflammatory	-	-	-
Hydroxychloroquine	36 (100.0)	9 (69.2)	-
Azathioprine	12 (33.3)	6 (46.2)	-
Mycophenolate mofetil	13 (36.1)	1 (7.7)	-
Methotrexate	4 (11.1)	7 (53.8)	-
Cyclosporine	1 (2.8)	2 (15.4)	-
Gammaglobulin	-	3 (23.1)	-
Disease activity parameter	Mean ± SD (range) or Number (%)		
SLEDAI-2K score	2.0 ± 2.7 (0–10)	-	-
SLEDAI-2K score ≥ 4	11 (30.6)	-	-
ERS (2 – 12 mm/h)	22.1 ± 13.4 (2.0 – 54.0)	16.9 ± 14.8 (3.0 – 50.0)	8.5 ± 3.2 (5.0 – 12.0)
CRP (< 5 mg/L)	1.9 ± 2.4 (0.3 – 9.8)	10.9 ± 14.8 (0.3 – 38.5)	0.3 ± 0.1 (0.3 – 0.6)
C3 (50 – 180 mg/dL)	98.8 ± 23.4 (50.0 – 141.0)	-	-
C4 (10 – 40 mg/dL)	15.3 ± 7.2 (5.8 – 35.1)	-	-
Anti-dsDNA (< 50 IU/mL)	84.7 ± 79.3 (2.5 – 200.1)	-	-
Anti-dsDNA +	23 (63.9)	-	-

ESR, erythrocyte sedimentation rate; CRP, C-reactive protein; C3, complement fraction 3; C4, complement fraction 4.
