# Supplementary material for: Reduced expressions of apoptosis-related proteins TRAIL, Bcl-2, and TNFR1 in NK cells of juvenile-onset systemic lupus erythematosus patients: relations with disease activity, nephritis, and neuropsychiatric involvement
Source: Front Immunol. 2024 Mar 18;15:1327255. doi: 10.3389/fimmu.2024.1327255 (PMC10982494; doi:10.3389/fimmu.2024.1327255)
Supplement: Supplementary file 2 [file DataSheet_2.docx]

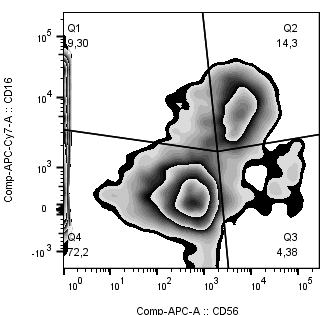

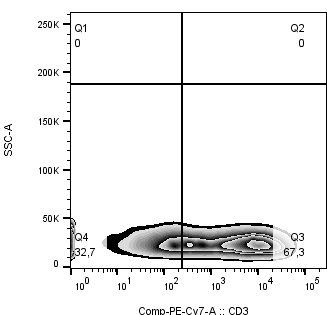

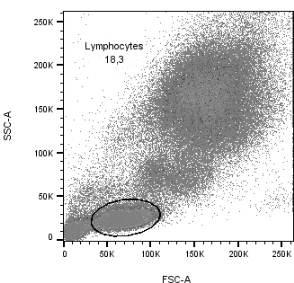


C

B

A


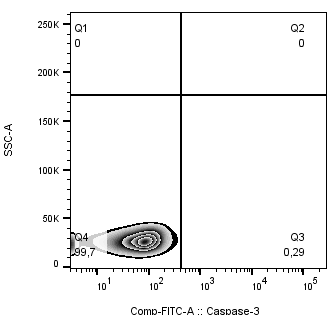

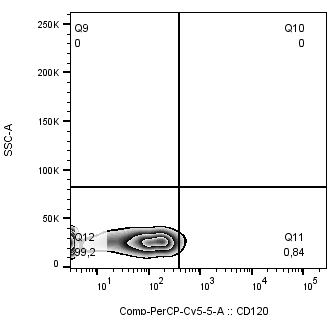

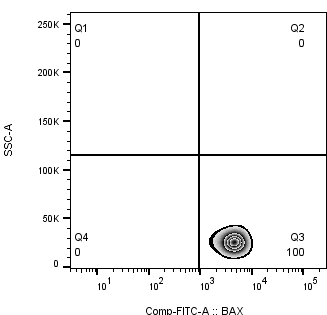

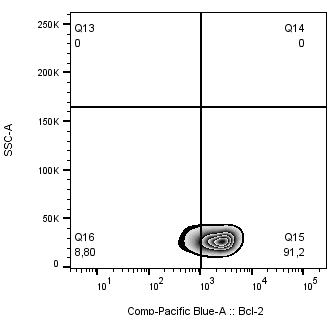

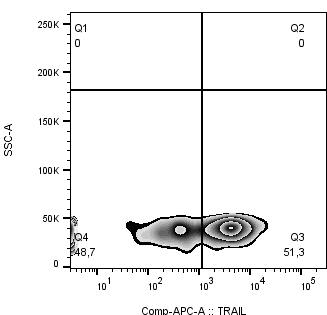

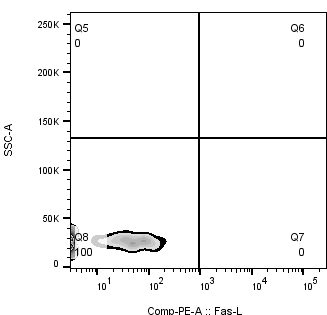

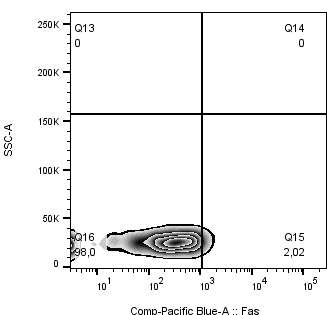

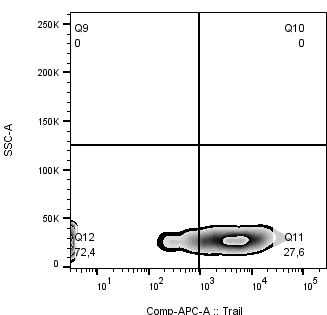

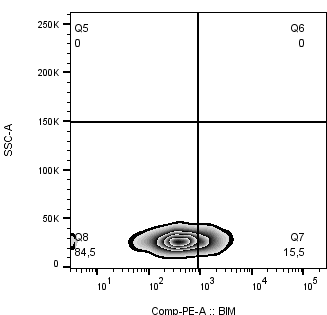

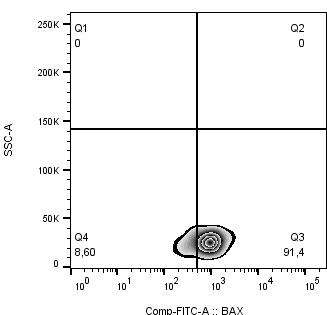

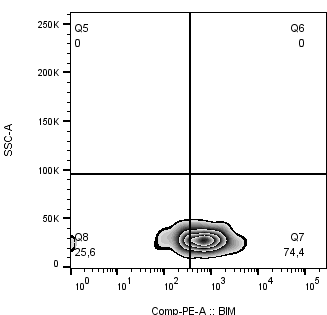

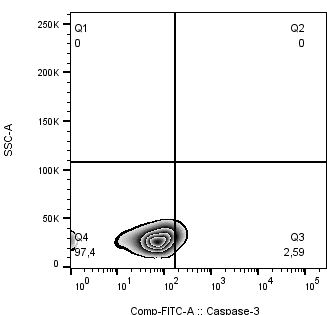

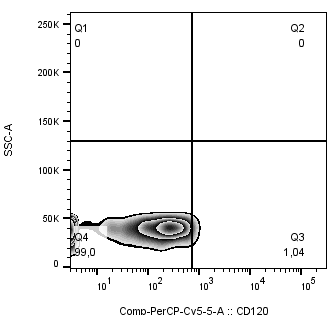

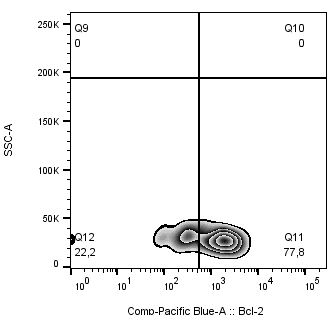

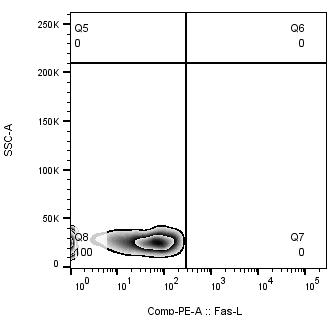

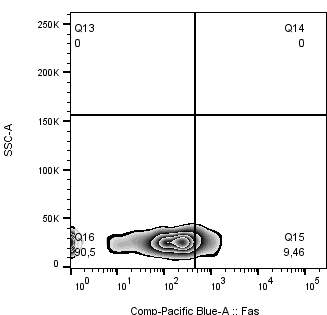

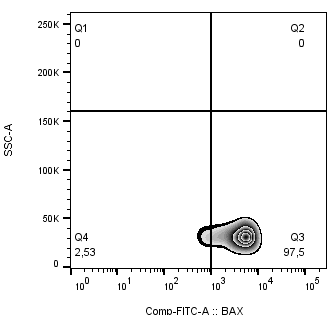

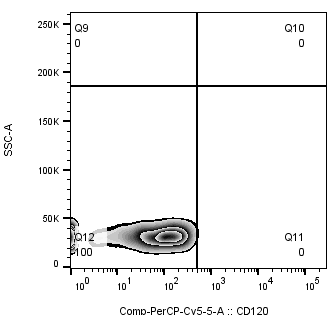

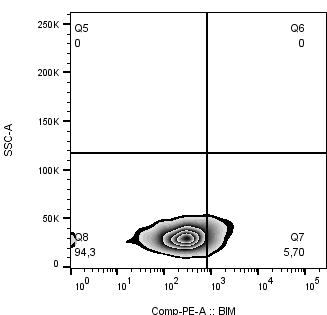

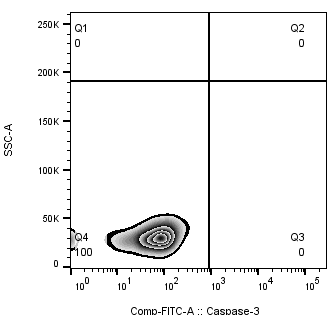

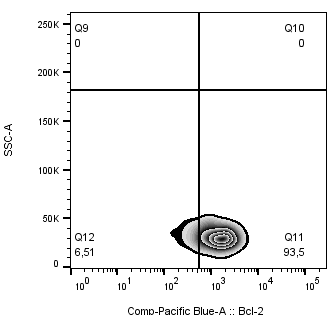

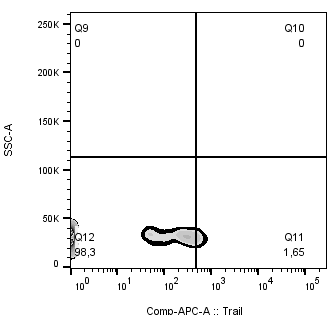

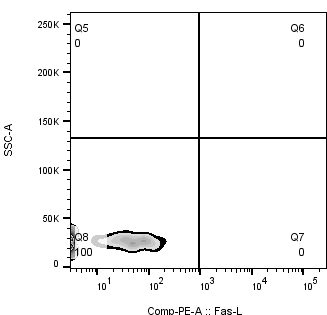

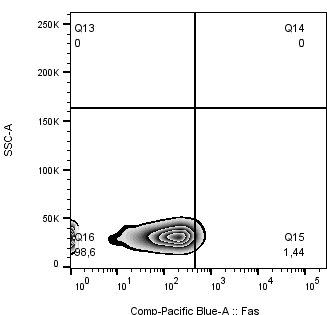


D

**Supplementary Figure** Representative gating strategy of NK cells analysis. Within lymphocyte population, based on forward and side scatter, CD3-negative cells were selected, and NK cells identified by double-positive CD16 and CD56 (A). Representative flow cytometry of each apoptosis-related protein expression in NK cells of juvenile-onset systemic lupus erythematosus patients (B), juvenile dermatomyositis controls (C), and healthy controls (D). Isotype controls of the same fluorochrome were included in all experiments.
